# Ovarian Precancerous Lesions and Their Molecular Landscape: A Structured Scoping Review Focused on STIC and Related Precursors of Epithelial Ovarian Cancer

**DOI:** 10.3390/cancers18183036

**Published:** 2026-09-18

**Authors:** Katarzyna Kwas-Sarnacka, Wiktoria Lisińska, Edward Koziróg, Kinga Stanek, Daniel Wolder, Maria Szubert, Jacek Wilczyński

**Affiliations:** 1 Department of Surgical and Oncologic Gynecology, 1st Department of Gynecology and Obstetrics, Medical University of Lodz, ul. Pomorska 251, 92-213 Lodz, Poland; 2 Department of Obstetrics and Gynecology, Jan Kochanowski University of Kielce, 25-369 Kielce, Poland; 3 Borahay’s Lab, Department of Gynecology and Obstetrics, Johs Hopkins Research Institute, Baltimore, MD 21205, USA

**Keywords:** ovarian cancer, precancerous lesions, high-grade serous ovarian cancer, endometriosis, borderline tumors

## Abstract

Ovarian cancer remains one of the deadliest gynecologic malignancies because of late-stage diagnosis, biological heterogeneity, and the lack of effective early detection strategies. Epithelial ovarian cancer (EOC) comprises several histotypes, including high-grade serous carcinoma (HGSC), low-grade serous carcinoma (LGSC), endometrioid ovarian carcinoma (ENOC), clear cell ovarian carcinoma (CCOC), and mucinous ovarian carcinoma (MOC), each with distinct molecular pathways and precursor lesions. In recent years, serous tubal intraepithelial carcinoma (STIC) has emerged as the principal precursor lesion of HGSC, supporting the fallopian tube origin paradigm of ovarian carcinogenesis. This structured scoping review summarizes current evidence on the molecular and cellular origins of epithelial ovarian cancer, with particular emphasis on precursor lesions, including *p53* signatures, SCOUTs, STILs, cortical inclusion cysts, endometriosis-associated lesions, and borderline ovarian tumors. Particular attention is given to the molecular events that drive progression toward invasive carcinoma, including *TP53* mutations, BRCA1/2 dysfunction, homologous recombination deficiency, chromosomal instability, telomere shortening, cell-cycle dysregulation, stemness-associated pathways, and inflammatory signaling. A structured literature search was conducted in PubMed/MEDLINE, Scopus, and Web of Science to identify studies published from 2000 to 2026 that addressed ovarian cancer precursor lesions, molecular pathogenesis, and early carcinogenic mechanisms. Articles were selected for relevance to epithelial ovarian carcinogenesis, precursor biology, and translational implications. Current evidence supports substantial biological heterogeneity among precursor lesions, particularly STICs, with only a subset likely to progress to invasive HGSC. Emerging molecular approaches, including genome-wide aneuploidy profiling and integrated genomic classifiers, may improve risk stratification and facilitate earlier detection of clinically significant precursor lesions. A better understanding of precursor biology may ultimately contribute to personalized prevention strategies and improved clinical outcomes in ovarian cancer.

## Simple Summary

Ovarian cancer is one of the most difficult gynecologic cancers to treat because it is often diagnosed only after it has spread. Many ovarian cancers develop from small, non-invasive changes in the fallopian tubes, ovaries, or surrounding tissues, but only some of these early lesions progress to cancer. Understanding which lesions are most likely to become malignant is essential for improving prevention, early diagnosis, and patient care. In this review, we summarize current knowledge about the earliest stages of epithelial ovarian cancer development, including the molecular changes that drive progression from precursor lesions to invasive disease. We also discuss emerging genomic approaches that may help identify high-risk lesions more accurately. A better understanding of these early events may support more personalized prevention strategies, improve early detection, and ultimately lead to better outcomes for women at risk of ovarian cancer.

## 1. Introduction

Ovarian cancer is one of the deadliest gynecological cancers. In developed countries, it is the fifth leading cause of cancer-related deaths among women [1,2]. The disease is usually diagnosed at a late stage (Stages III and IV), possibly due to a lack of specific symptoms and effective methods of early detection [2].

It is estimated that there were over 320,000 new cases and over 200,000 deaths from ovarian cancer globally in 2022, highlighting the scale of the health problem [3]. Established risk factors for epithelial ovarian cancer include age, hereditary mutations (BRCA1/2, RAD51C/D, BRIP1, Lynch syndrome genes), nulliparity, infertility, endometriosis, obesity, hormone therapy, and prolonged ovulation. Survival varies markedly by stage and histological subtype. Five-year survival exceeds 90% in stage I disease but falls below 40% in stage III and 20% in stage IV. HGSC has the poorest prognosis due to late-stage presentation, whereas LGSC is associated with longer survival despite chemoresistance. Early-stage ENOC and CCOC generally have favorable outcomes, although advanced CCOC responds poorly to platinum-based chemotherapy. MOC shows excellent survival when localized but poor outcomes in metastatic disease [1,2,3,4].

Unfortunately, most cases of ovarian cancer are still diagnosed too late, and available treatments are not very effective [2,3]. Consequently, improving our understanding of ovarian cancer precursor lesions has become a major research priority. Characterization of these early lesions may not only provide insight into ovarian carcinogenesis but also facilitate the development of more effective prevention and early detection strategies [4]. Given the marked molecular and histopathological heterogeneity of epithelial ovarian cancer, precursor lesions are likewise believed to be histotype-specific rather than universal.

The strongest evidence pertains to high-grade serous carcinoma (HGSC), which primarily develops in the fallopian tube epithelium [5]. Lesions known as serous tubal intraepithelial carcinoma (STIC) exhibit the same *TP53* mutations as coexisting HGSC tumors, suggesting a clonal origin and confirming their significance as preinvasive lesions [5,6]. In the case of ENOC and CCOC, there is strong evidence that endometriosis is a precursor lesion [7]. *ARID1A* gene mutations and loss of BAF250a protein expression have been shown to be present in foci of atypical endometriosis next to the tumor. This suggests that these mutations may be an early molecular event in EAOC carcinogenesis [7,8].

**Figure 1:**
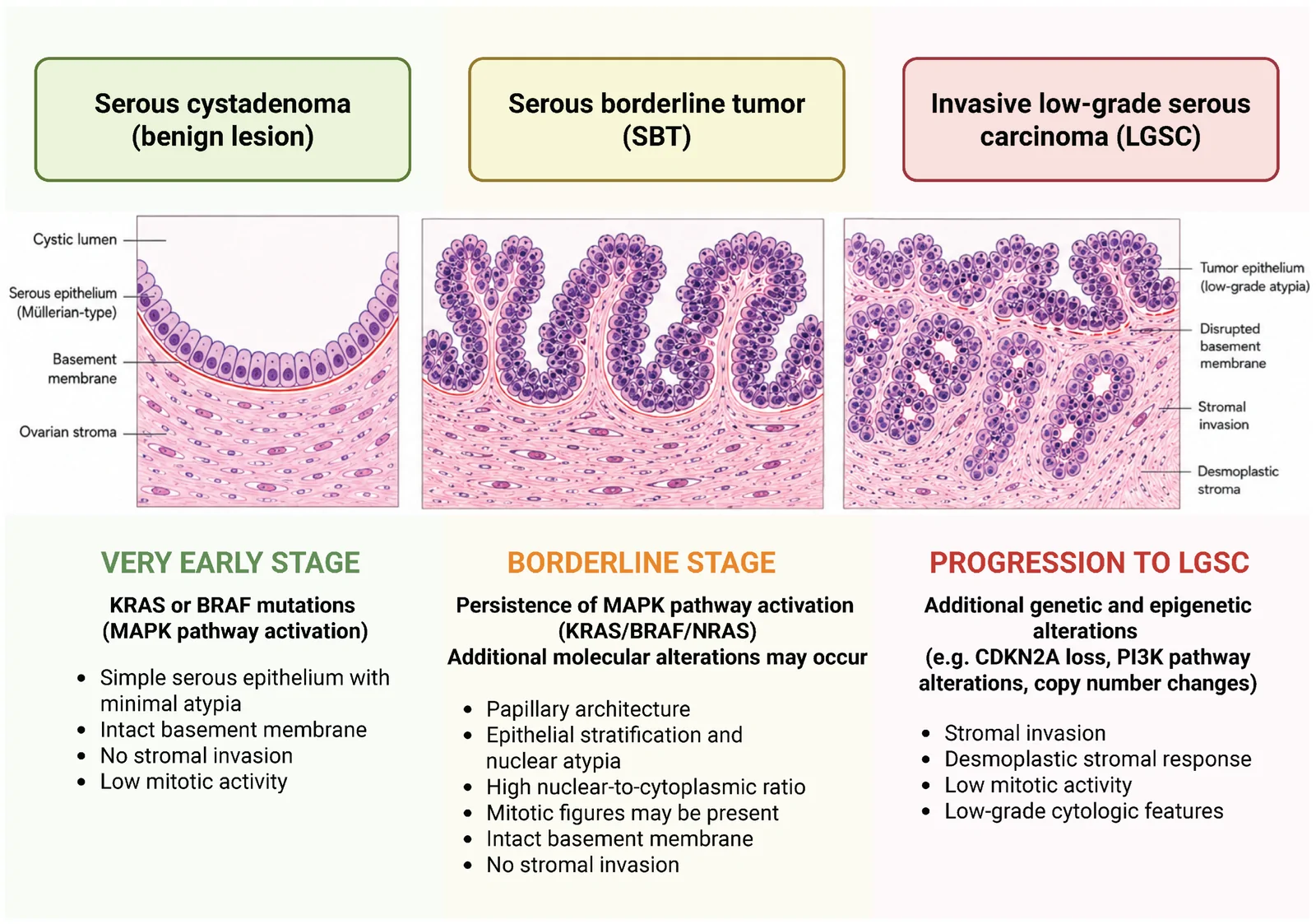
Proposed stepwise progression model of low-grade serous carcinoma (LGSC). The figure illustrates the transition from benign serous lesions through serous borderline tumors (SBTs) to invasive LGSC. Activating alterations in the MAPK pathway, particularly involving KRAS, BRAF, and NRAS, represent the major recurrent molecular events in LGSC and are also observed in serous borderline tumors, supporting a molecular continuum underlying LGSC development. Other genomic alterations have been reported at lower frequencies, but they are not considered defining molecular drivers of conventional LGSC. The lower panel schematically depicts the evolution from normal epithelium to invasive cancer through clonal expansion and increasing genetic complexity. Abbreviations: LGSC, low-grade serous carcinoma; SBT, serous borderline tumor; KRAS, Kirsten rat sarcoma viral oncogene homolog; BRAF, B-Raf proto-oncogene, serine/threonine kinase; NRAS, neuroblastoma RAS viral oncogene homolog. Created with BioRender.

LGSC, on the other hand, is considered a tumor that develops through stepwise progression from benign lesions to serous borderline tumors (SBTs) and then to invasive cancer [9]. This process is supported by the presence of *KRAS* and *BRAF* mutations in both SBT and LGSC, suggesting a well-defined precursor continuum [9,10]. This proposed stepwise progression is illustrated in Figure 1.

## 2. Aim of the Study

This manuscript aims to summarize the latest developments in our understanding of ovarian cancer precursor lesions, distinguishing between concepts supported by strong evidence and those that remain hypothetical. Given the rapidly expanding body of literature on ovarian cancer precursor lesions, a scoping review approach was considered appropriate to systematically map the available evidence across different precursor pathways and molecular mechanisms. Accordingly, the literature was identified and selected using a structured scoping review methodology. References were selected based on predefined eligibility criteria and their relevance to three main areas: (i) the most recent advances in the field, (ii) well-established evidence, and (iii) emerging hypotheses related to each proposed precursor pathway.

## 3. Methods

### 3.1. Review Design and Objective

This study was conducted and reported in accordance with the PRISMA 2020 guidance (Table S1). This study was conducted as a structured narrative review focused on the molecular and cellular mechanisms underlying precursor lesions of epithelial ovarian cancer. The primary objective was to summarize current evidence regarding the pathogenesis of ovarian cancer precursor lesions, with particular emphasis on serous tubal intraepithelial carcinoma (STIC) and related molecular alterations associated with progression toward high-grade serous carcinoma (HGSC).

### 3.2. Literature Search Strategy

A comprehensive literature search was conducted using PubMed/MEDLINE, Scopus, and Web of Science databases to identify relevant articles published between January 2000 and March 2025. The search strategy included combinations of Medical Subject Headings (MeSH) and free-text keywords such as: “ovarian cancer”, “high-grade serous carcinoma”, “serous tubal intraepithelial carcinoma”, “STIC”, “fallopian tube carcinogenesis”, “p53 signature”, “SCOUT”, “STIL”, “TP53”, “endometriosis-associated ovarian cancer”, “borderline ovarian tumors”, “cortical inclusion cysts”, “genomic instability”, and “aneuploidy”. Boolean operators (“AND”, “OR”) were applied to optimize the search strategy.

### 3.3. Eligibility Criteria and Data Synthesis

Studies were included if they addressed molecular, histopathological, or translational aspects of ovarian cancer precursor lesions and epithelial ovarian carcinogenesis. Original articles, translational studies, genomic analyses, clinicopathological studies, and relevant review articles published in peer-reviewed journals in English were considered eligible. Conference abstracts without full-text availability, isolated case reports without mechanistic relevance, and non-English publications were excluded.

The selected literature was narratively synthesized and organized into thematic sections focusing on: (1) the fallopian tube origin of HGSC, (2) molecular progression from p53 signatures to STIC, (3) genomic instability and homologous recombination defects, (4) inclusion cysts and alternative precursor pathways, (5) endometriosis-associated carcinogenesis, (6) borderline ovarian tumors and low-grade serous pathways, and (7) clinical implications and future translational perspectives. Particular emphasis was placed on studies with substantial molecular, translational, or clinicopathological relevance, including landmark investigations that shaped the contemporary understanding of ovarian carcinogenesis.

The review question was conceptually structured according to PICO-based principles adapted for mechanistic and translational oncology research. A formal assessment of the certainty of evidence was not performed because of the heterogeneous nature of the included literature and the narrative design of the review.

### 3.4. Data Collection Process

Data were extracted from eligible publications identified through the literature search and subsequently assessed in full text. Two reviewers independently screened the titles and abstracts and assessed potentially eligible articles in full text. Relevant information was collected with particular attention to molecular, histopathological, genomic, and translational findings concerning ovarian cancer precursor lesions, with emphasis on STIC and its progression toward HGSC. The extracted information was organized according to the main thematic areas of the review, including the fallopian tube origin of HGSC, progression from *p53* signatures to STIC, genomic instability and homologous recombination defects, alternative precursor lesions, endometriosis-associated carcinogenesis, borderline ovarian tumors, and clinical implications. The review protocol did not specify a standardized data-extraction form or dedicated electronic extraction tool. The individual reviewers responsible for the final extraction of data from each included publication were also not explicitly identified. Reviewers resolved discrepancies regarding study eligibility or interpretation through discussion and consensus.

### 3.5. Study Risk of Bias Assessment

A formal risk-of-bias assessment was not performed because the included literature was heterogeneous and predominantly mechanistic, histopathological, genomic, and translational. The review incorporated different types of publications, including original research, translational studies, genomic analyses, clinicopathological studies, and relevant review articles, which limited the applicability of a single standardized risk-of-bias assessment tool across all included evidence. Instead, we interpreted the available evidence based on each study’s relevance to the review question, its methodological and biological context, and the consistency of findings across the literature. This methodological limitation was considered when synthesizing and interpreting the evidence.

### 3.6. Reporting Bias Assessment

Publication bias was not formally assessed because no quantitative meta-analysis was performed. Given the heterogeneous and predominantly mechanistic, histopathological, genomic, and translational nature of the included literature, a formal assessment of publication or reporting bias was not considered applicable to the present structured narrative review.

### 3.7. Study Selection

**Figure 2:**
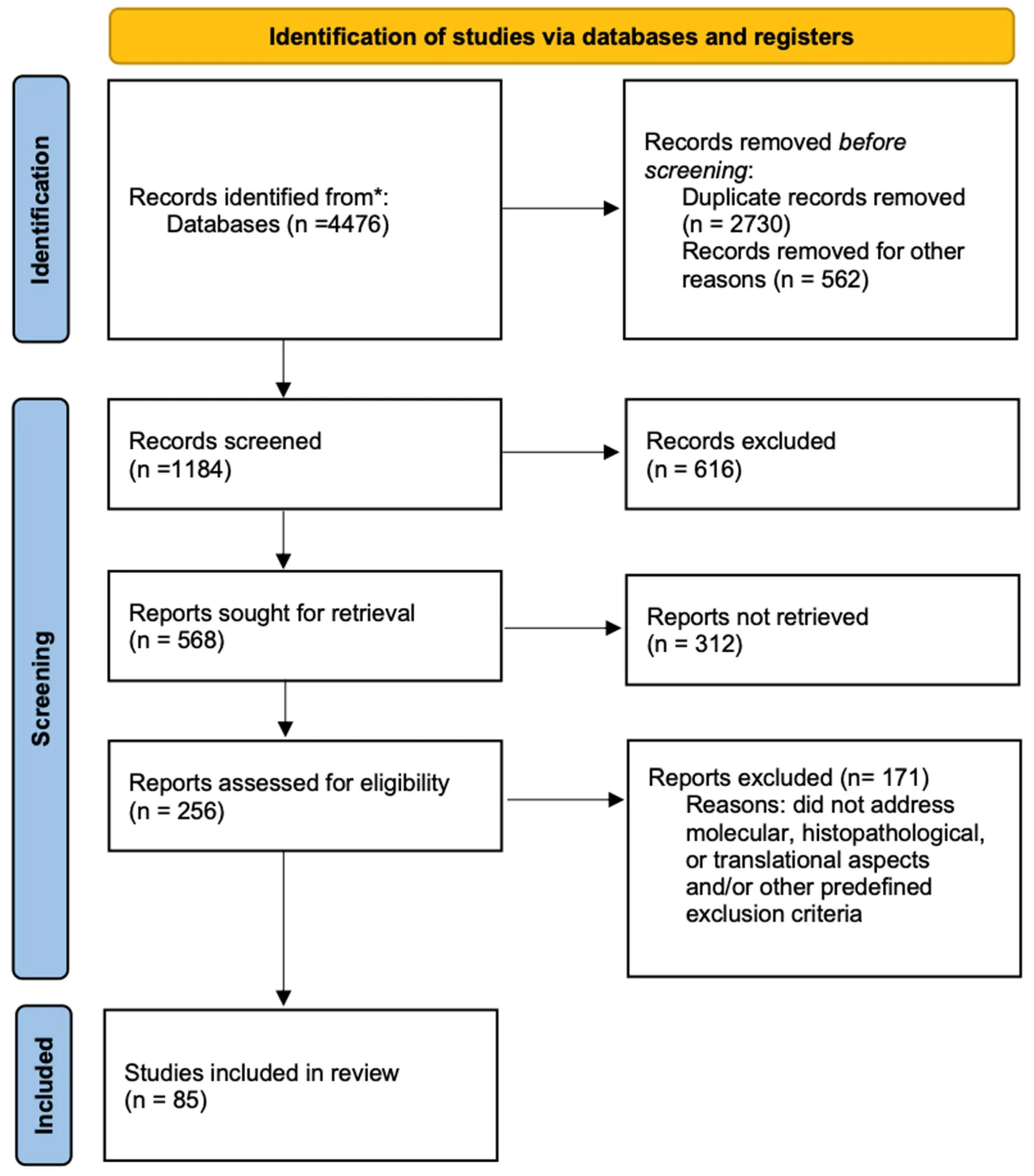
PRISMA flow diagram.

Following the predefined search strategy, we screened all records identified from PubMed/MEDLINE, Scopus, and Web of Science for relevance to the review objective. We removed duplicate records before title and abstract screening. Potentially relevant articles were subsequently assessed in full text according to the predefined inclusion and exclusion criteria. Articles were excluded when they did not address the molecular, histopathological, or translational aspects of ovarian cancer precursor lesions, or when they met other predefined exclusion criteria. A total of (*n* = 85) studies were ultimately included in the qualitative narrative synthesis. The detailed study selection process, including the numbers of records identified, screened, excluded, assessed in full text, and included, is presented in the PRISMA 2020 flow diagram below in Figure 2 [10].

## 4. Tubal Paradigm of High-Grade Serous Carcinoma (HGSC)

The pathogenesis of HGSC is driven by cumulative genotoxic stress, including oxidative, inflammatory, and hormonal insults, with ovulation acting as a recurrent, localized source of reactive oxygen species, cytokines, and growth factors that directly damage fimbrial epithelial DNA [11,12,13]. This damage, coupled with *TP53* mutations and subsequent genomic instability, promotes clonal expansion of p53-signature cells [6,14]. Follicular fluid–mediated activation of inflammatory and oncogenic pathways, including the NF-κB–miR-155 axis, fosters acquisition of stem-like traits in precursor lesions [12,15]. Follicular fluid is rich in bioactive molecules such as reactive oxygen species, steroid hormones, prostaglandins, and cytokines (e.g., IL-6, IL-8), which can induce DNA damage and activate pro-survival signaling pathways in fallopian tube epithelial cells. In addition, it has been shown to stimulate key oncogenic cascades, including PI3K/AKT and MAPK signaling, thereby enhancing cellular proliferation, resistance to apoptosis, and genomic instability. These effects collectively create a microenvironment conducive to early malignant transformation and clonal selection of genetically altered cells [12,13,14,15]. Retrograde menstruation further amplifies oxidative and pro-inflammatory stress through iron-mediated DNA damage and cytokine exposure [16]. Subsequent molecular alterations, including *BRCA1/2* loss, *CCNE1* amplification, and telomere shortening, contribute to the progression from p53 signatures to STIC. STIC is characterized by nuclear enlargement, hyperchromasia, loss of polarity, increased Ki-67 expression, and γH2AX positivity. These changes reflect increasing genomic instability and proliferative activity during the transition toward HGSC [5,11,17]. Exfoliated STIC cells have been proposed to disseminate to the ovarian or peritoneal surfaces, a model referred to as “precursor escape.” Although this mechanism provides a plausible explanation for some cases of apparently ovarian HGSC with a tubal precursor, its frequency and contribution relative to other routes of tumor dissemination remain uncertain [18]. Collectively, repeated cycles of epithelial injury and repair create a permissive evolutionary landscape in which genetically altered fimbrial cells evolve into STIC and ultimately HGSC [12,14]. Evidence supporting this model includes the frequent identification of STIC lesions in prophylactic salpingo-oophorectomy specimens from *BRCA1/2* mutation carriers, shared molecular alterations (*TP53* mutations, *BRCA* loss) between STIC and HGSC, and the spatial predilection of lesions to the distal fimbriae [5,6,10,11,15].

An important unresolved question is why only a subset of STIC lesions appears to progress to invasive HGSC. TP53 mutation alone is unlikely to determine progression, as p53 signatures and STICs may persist without immediate invasive transformation. Additional factors may include the extent of chromosomal instability, telomere dysfunction, defects in homologous recombination, cell-cycle dysregulation, epigenetic alterations, and interactions with the local immune and stromal microenvironment. Recent genomic studies suggest that aggressive precursor lesions may be distinguished by patterns of aneuploidy and broader genomic instability rather than by TP53 status alone. However, most available evidence is derived from cross-sectional or retrospective studies, and longitudinal data linking specific molecular features in STIC to subsequent invasive transformation remain limited. Consequently, the molecular features that define a truly high-risk STIC remain incompletely established.

Although STIC is widely accepted as an important precursor lesion for many HGSCs, the exact proportion of tumors arising through the STIC–HGSC pathway remains uncertain. Current estimates suggest that a substantial proportion of HGSCs are associated with STIC lesions; however, not all STICs necessarily progress to invasive carcinoma [11,19]. Nevertheless, several studies suggest that a subset of HGSCs may arise through alternative pathways, including precursor escape mechanisms, cortical inclusion cysts containing tubal-type epithelium, or lesions that remain below current histopathological detection thresholds. Therefore, the STIC–HGSC sequence should be viewed as the dominant, but not exclusive, model of HGSC development [10,11,19].

### 4.1. STIC (Serous Tubal Intraepithelial Carcinoma) in the Tubal Paradigm of HGSC

The earliest identifiable molecular alteration in the tubal epithelium is the *p53* signature [6,14]. These lesions are histologically normal secretory cells exhibiting *TP53* mutations and positive γH2AX staining, a marker of DNA double-strand breaks, but show minimal proliferation (Ki-67 < 10%) [6,20]. Although fallopian tube secretory cells are widely considered a major candidate cell population for HGSC initiation, recent experimental studies suggest that progenitor and pre-ciliated epithelial cell populations may also contribute to tumor development. These findings indicate greater cellular heterogeneity of HGSC origin than previously recognized and suggest that the precise cell of origin remains incompletely defined. Morphological changes are absent, underscoring the need for immunohistochemistry for diagnosis. *p53* signatures can be bilateral, multifocal, and persistent without progression, representing a pool of genetically altered yet largely quiescent epithelial clones [20,21]. Importantly, *TP53* mutation alone is insufficient to drive malignant transformation, as evidenced by the presence of *p53* foci in healthy *BRCA* mutation carriers and Li–Fraumeni syndrome patients without ovarian malignancy. Progression from *p53* signature to invasive disease requires additional genetic, epigenetic, and environmental insults over time [14,21].

### 4.2. Secretory Cell Expansion (SCE) and Secretory Cell Outgrowths (SCOUTs)

Secretory cell expansion (SCE) refers to a localized increase in the number of morphologically bland secretory epithelial cells within the fallopian tube. Unlike STIC, SCE is not characterized by marked cytologic atypia or an established malignant phenotype. Its molecular characteristics and relationship to TP53-mutant precursor lesions remain incompletely defined. Therefore, SCE is currently best regarded as a distinct epithelial alteration of uncertain biological significance rather than an established precursor lesion of HGSC.

SCOUT lesions represent early proliferative expansions of at least 30 secretory cells within the fallopian tube epithelium. They are characterized by BCL2 positivity, p53 negativity, and loss of PAX2, a feature also observed in STIC and HGSC [22,23]. SCOUTs increase in frequency with age, but their temporal relationship to p53 signatures and STIC remains uncertain. Their distinct anatomical distribution and molecular features suggest that they may represent a biologically distinct secretory-cell alteration rather than an obligatory intermediate stage in the canonical STIC–HGSC pathway. However, due to their unclear clinical significance, SCOUTs are rarely reported in routine histopathology, and their natural history remains poorly defined [22,23,24]. An important distinction between these precursor lesions is their anatomical distribution along the fallopian tube. p53 signatures and STICs show a strong predilection for the distal, fimbriated portion of the tube, whereas SCOUTs are more broadly distributed and are frequently identified in more proximal regions. In one comparative study, 85% of STICs and 62% of p53 signatures were located in the fimbrial region, while SCOUTs were predominantly found in the infundibulum (65%) and ampulla (26%). Notably, no STICs were identified proximal to the infundibulum in that series, whereas p53 signatures were occasionally detected in the ampulla. These distinct anatomical distributions support the concept that SCOUTs and p53 signatures/STICs may represent biologically distinct lesions rather than sequential stages of a single linear pathway [22,24,25,26].

### 4.3. Serous Tubal Intraepithelial Lesions (STILs) and Serous Tubal Intraepithelial Carcinomas (STICs)

STILs exhibit mild cytologic atypia that exceeds that of typical p53 signatures but does not meet the full morphological criteria for STIC. Although they may represent an intermediate morphological phenotype, their temporal position within the HGSC precursor spectrum and their independent precursor significance remain uncertain.

These lesions harbor early *TP53* mutations and minor chromosomal alterations [24,25]. Their proliferative activity is variable, and the risk of progression to STIC or HGSC is currently undetermined. STILs underscore the biological heterogeneity of tubal precursor lesions and highlight the importance of molecular as well as histopathological characterization in risk assessment [24,25,26].

**Figure 3:**
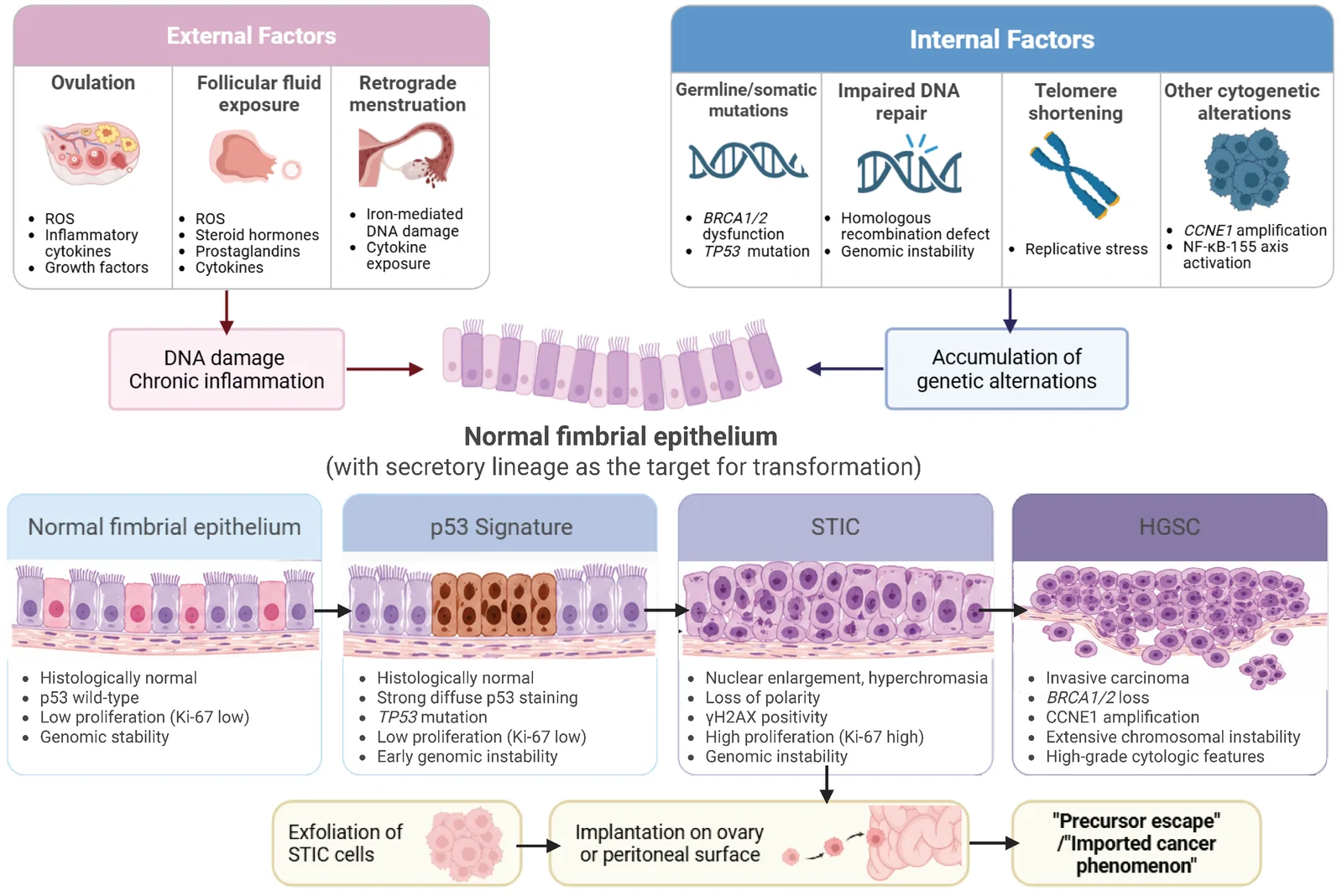
Proposed model of fallopian tube-associated HGSC development. The figure illustrates the currently supported association between normal fimbrial epithelium, p53 signatures, STIC, and invasive HGSC. Although this sequence represents the best-characterized pathway, not all precursor lesions necessarily progress to invasive carcinoma, and alternative or complementary mechanisms of HGSC development have also been proposed. Created in Biorender.

The proposed sequence of fallopian tube precursor lesions and their progression toward HGSC is illustrated in Figure 3.

STIC represents an important precursor state within the fallopian tube-associated pathway of HGSC development, although its biological heterogeneity and relationship to established HGSC require careful interpretation. Morphologically, STIC is defined by nuclear enlargement, hyperchromasia, prominent nucleoli, loss of epithelial polarity, and reduction or absence of ciliated cells [6,11]. Mitotic figures are often abundant, and the lesion remains confined above the basement membrane. Diagnostic criteria include strong *p53* immunostaining (>60%) or complete absence (null pattern) and an elevated proliferation index (Ki-67 > 10%). STICs are most frequently located in the distal fimbriae, representing the majority of precursor lesions in prophylactic salpingo-oophorectomy specimens from high-risk women [11,27,28].

Molecularly, STICs show complex alterations indicative of early malignant transformation. *TP53* mutations are nearly universal, serving as the foundational genomic event, often accompanied by chromosomal instability and partial or complete loss of chromosome 17, which affects both *TP53* and *BRCA1*. *BRCA1* loss is a critical modifier in carriers, impairing homologous recombination-mediated DNA repair and promoting genomic instability [11,17,27,29]. Additional molecular hallmarks include telomere shortening, stem cell marker expression (SOX2, ALDH1A1), and, in some cases, *CCNE1* amplification or *RB1* loss, which drive proliferation and cell cycle dysregulation. Notably, these molecular changes occur even when the lesion appears morphologically subtle, underscoring the limitations of histopathology alone for risk prediction [11,15]. Clinically, STICs are pivotal for early detection and risk stratification. Approximately 60–80% of HGSCs are reported to be associated with STIC lesions, supporting their role as an important precursor in many cases. However, the biological behavior of STIC appears heterogeneous, and not all lesions necessarily progress to invasive carcinoma [28,30]. However, STICs rarely occur in isolation; they are often found adjacent to or intermixed with invasive HGSC, complicating clonal interpretation. Importantly, STIC-like lesions may also represent secondary involvement of the fallopian tube by an established primary ovarian HGSC rather than a true tubal precursor lesion. Such lesions may result from implantation or spread of malignant cells to the tubal epithelium and can therefore mimic primary STIC. This possibility highlights the importance of careful morphological and molecular assessment when interpreting STIC-like lesions, particularly in cases with an established ovarian mass. Furthermore, a subset of STIC lesions may remain biologically indolent and fail to progress to invasive carcinoma. Whether this reflects true cellular dormancy, senescence, immune-mediated suppression, or other mechanisms remains uncertain. These observations suggest that STIC is biologically heterogeneous and that morphological diagnosis alone may be insufficient to predict which lesions have clinically relevant malignant potential. An important diagnostic caveat is that STIC-like lesions identified in the presence of an established pelvic HGSC should not automatically be interpreted as the primary precursor lesion. In some cases, intraepithelial tubal involvement may represent secondary or metastatic spread of an already established carcinoma. Therefore, the distinction between true precursor STIC and secondary tubal involvement requires careful integration of morphology, anatomical distribution, and molecular clonality. The temporal progression from initial DNA damage to STIC formation is estimated at 15 years, with additional years required for invasion and ovarian implantation, providing a potential window for early detection and preventive interventions [28,30,31]. From an evolutionary perspective, STIC represents a critical bottleneck in HGSC pathogenesis. While some lesions may remain dormant due to telomere crisis, oncogene-induced senescence, or immune-mediated suppression, others acquire additional alterations that enable proliferation, invasion, and metastasis [11,15,28,30,32].

**Figure 4:**
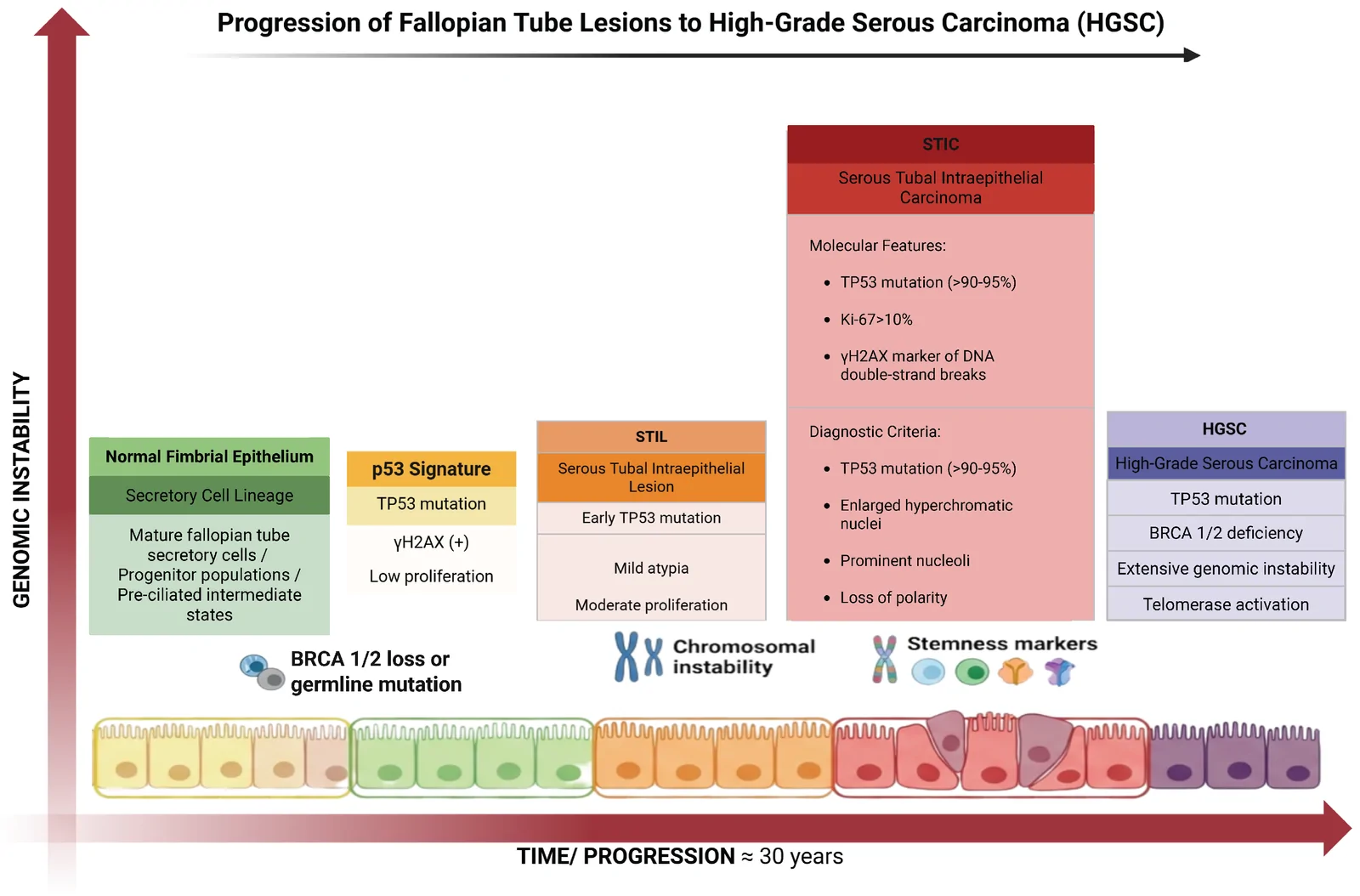
Progression of Fallopian Tube Lesions to High-Grade Serous Carcinoma (HGSC). *ALDH1A1*: Aldehyde Dehydrogenase 1 Family Member A1; *BRCA1/2*: Breast Cancer Type 1/2 Susceptibility Genes; *CCNE1*: Cyclin E1; FTE: Fallopian Tube Epithelium; HGSC: High-Grade Serous Carcinoma; Ki-67: Proliferation Marker (nuclear antigen); PAX2: Paired Box Gene 2; SCOUT: Secretory Cell Outgrowth; *SOX2*: *SRY-Box* Transcription Factor 2; STIC: Serous Tubal Intraepithelial Carcinoma. Created in Biorender.

To sum up, HGSC represents the invasive endpoint of the tubal carcinogenesis cascade. Cells originating from STIC or exfoliated fimbrial precursors may implant on the ovarian surface or within cortical inclusion cysts, where the ovarian microenvironment promotes malignant transformation. HGSC exhibits invasive architecture, high proliferative activity, extensive chromosomal instability, *TP53* and *BRCA1/2* mutations, telomerase activation, and stem cell marker expression. While STICs account for the majority (~60–80%) of HGSCs, the remainder may arise from direct transformation within ovarian surface epithelium, cortical inclusion cysts, or, rarely, low-grade serous tumors [31,32,33,34]. The proposed progression from normal fimbriae epithelium through p53 signature and STIC to invasive HGSC is illustrated in Figure 4.

Taken together, these lesions represent a heterogeneous spectrum of fallopian tube epithelial alterations rather than a uniformly sequential pathway. p53 signatures and STICs are the best-characterized lesions within the HGSC pathway, whereas the biological significance and temporal relationships of SCE, SCOUTs, and STILs remain less certain. Their distinction requires integration of morphology, immunophenotype, molecular findings, and anatomical distribution (Table 1).

## 5. Molecular Mechanisms Driving HGSC Progression of Ovarian Cancer

### 5.1. TP53 Mutation as the Foundational Genetic Event

The defining molecular hallmark of STIC is mutation of the *TP53* tumor suppressor gene, which occurs in over 95% of HGSCs and represents the earliest genetic event in tumorigenesis. Given the high prevalence of *TP53* mutations in HGSC, signaling pathways regulated by this tumor suppressor are clearly essential for the initial cellular alterations that ultimately lead to malignant transformation. Loss of *p53* function permits accumulation of DNA damage, circumvention of cell-cycle checkpoints, and survival of genetically unstable cells, while certain oncomorphic *TP53* mutations may actively promote tumorigenesis [35,36].

Genomic and spatial studies support increasing molecular complexity from p53 signatures to STIC and ultimately to HGSC, while also revealing substantial heterogeneity in the genomic and immune microenvironment of precursor lesions. Whereas p53 signatures typically harbor isolated TP53 mutations with few additional genomic alterations, STICs exhibit higher mutational burdens, chromosomal instability, and loss of heterozygosity (LOH), reflecting increasing molecular complexity during precursor progression [37,38,39,40,41]. Recent spatial and multiomic studies further demonstrate substantial molecular heterogeneity among ovarian precursor lesions, including differences in aneuploidy, BRCA1/2 alterations, CCNE1/MYC amplification, proliferative activity, and immune-regulatory programs [42,43].

Multiple *p53* signatures and STIC lesions often occur in the same individual—especially in *BRCA*1/2 mutation carriers—and are usually clonally independent, supporting parallel rather than linear evolution. Repeated exposure to carcinogenic factors may generate many altered epithelial clones, but only a few progress to HGSC [44,45].

Spatial integration of protein and chromosomal states has additionally identified early copy-number alterations and genotype-associated immune neighborhoods during serous ovarian cancer evolution, providing further evidence that genomic and microenvironmental changes emerge early during disease progression [46,47].

The prognostic value of *p53* signatures remains unclear, as similar frequencies are found in both healthy and *BRCA*-mutated individuals. However, tubes with STICs show more *p53* signatures and a higher prevalence of *TP53* mutations [41,44].

Overall, *TP53* inactivation is necessary but not sufficient for malignant transformation, requiring additional genetic and environmental events [45].

Experimental studies further support the proposed role of the fallopian tube in HGSC initiation. Exposure of human fallopian tube epithelium to follicular fluid induces molecular and phenotypic changes resembling early serous carcinogenesis [13,41], while experimental studies of the ovarian cortical inclusion cyst microenvironment demonstrate that extracellular matrix composition can modulate the invasive behavior of fallopian tube epithelial cells [47,48,49,50,51]. These findings provide functional support for a role of both tubal epithelial alterations and the local microenvironment in early serous carcinogenesis.

### 5.2. Homologous Recombination Deficiency and BRCA-Associated Carcinogenesis

In *BRCA1* germline mutation carriers, progression from early tubal epithelial alterations to STIC is facilitated by profound defects in homologous recombination-mediated DNA repair, which synergize with loss of *p53* function to promote genomic instability [41]. This cooperative impairment markedly increases the risk of HGSC, positioning *BRCA1* deficiency as a key modifier of STIC evolution rather than a sole initiating event. Accordingly, the near-universal presence of *TP53* mutations in HGSC should not be interpreted as evidence of direct causality in disease initiation, but rather as a conserved and selectively advantageous molecular alteration retained during tumor evolution [47]. Because most HGSCs are diagnosed late, tumor genotyping cannot determine the order of mutations. However, abnormal *p53* in normal fallopian tube tissue suggests the tubal epithelium enables cancer development, with STIC as a key intermediate stage. Of particular interest is the recurrent involvement of chromosome 17, which harbors both *TP53* and *BRCA1*. Partial or complete loss of chromosome 17 provides a plausible mechanistic link between early aneuploidy and the simultaneous inactivation of these two critical tumor suppressors, offering new insight into why *BRCA1*, but not *BRCA2*, germline mutations confer a markedly elevated risk of HGSC [18,41,48]. This observation supports a model in which chromosomal instability precedes or accelerates key driver events, positioning aneuploidy as a potential upstream determinant of aggressive STIC behavior rather than a late consequence of transformation.

*BRCA1*- and *BRCA2*-deficient tumors differ in mutational burden and clinical behavior, reflecting their distinct roles in the DNA damage response. Studies comparing fallopian tube epithelial cells from BRCA1 and BRCA2 mutation carriers revealed distinct molecular profiles. BRCA1 deficiency is associated with increased inflammatory signaling, altered DNA damage responses, and enhanced proliferation, whereas BRCA2 deficiency mainly impairs homologous recombination repair, suggesting different pathways of HGSC development.

Molecular profiling studies further show that the fallopian tube epithelium of *BRCA1* mutation carriers exhibits altered transcriptional programs compared with controls, particularly affecting inflammatory signaling, stress responses, and DNA damage pathways [46]. Although cyclic hormonal fluctuations induce broad changes in gene expression in the tubal epithelium irrespective of genotype, *BRCA1*-mutant tissue exhibits a distinct inflammatory and damage-response signature, including upregulation of CEBP-δ, NAMPT, and GADD45β, alongside reduced STAT3 activation. These alterations likely modify epithelial responses to environmental stressors, increasing the likelihood of malignant transformation over time [49].

### 5.3. Cell Cycle Dysregulation

*CCNE1* (cyclin E1) amplification and *RB1* loss disrupt cell cycle control, promoting centrosome amplification, chromosomal instability, and chemoresistance. These alterations are typically mutually exclusive with *BRCA1/2* loss and are associated with poor prognosis. Although recurrent point mutations beyond *TP53* are uncommon in STICs, defects in DNA damage repair pathways are frequently implicated in progression. Germline or somatic alterations in homologous recombination (HR) genes, particularly *BRCA*1 and *BRCA*2, accelerate the transition from early precursor lesions to STIC and HGSC [31,50]. In HR-proficient tumors, *CCNE1* amplification is believed to represent an alternative oncogenic mechanism that may contribute to genomic instability and cell-cycle dysregulation. Evidence suggests that *CCNE1* amplification may arise during or shortly after the STIC stage, conferring a strong proliferative advantage and facilitating rapid progression [48].

### 5.4. Telomere Dysfunction and Replicative Stress

Telomere shortening is a prominent feature of STICs and offers further insight into their evolutionary status. STICs, particularly those associated with HGSC, often have the shortest telomeres among tubal lesions, reflecting extensive replicative history and genomic stress. Critically short telomeres promote chromosomal instability, centrosome amplification, and DNA breakage, all of which are observed in STICs. Although such instability can trigger apoptosis or senescence in many lesions, a subset of STICs appears to activate telomerase, thereby maintaining telomeres and escaping crisis. This event likely marks a key transition toward invasive HGSC [32,52]. STICs show marked heterogeneity in proliferative activity, as assessed by Ki-67 labeling indices. While many STICs display high proliferative rates consistent with aggressive behavior, a substantial subset shows low proliferative activity comparable to that of normal epithelium. These lesions, termed “dormant STICs,” raise critical questions about their biological potential. Dormant STICs may represent evolutionary dead ends resulting from telomere crisis, oncogene-induced senescence, or immune-mediated suppression [53]. Alternatively, they may retain the capacity to re-enter the cell cycle upon acquiring additional genetic or epigenetic alterations, thereby contributing to delayed tumorigenesis [54].

### 5.5. Autophagy and Cancer Stem Cell Biology

Altered autophagy pathways, including BECN1 haploinsufficiency, contribute to tumor initiation and genomic instability. Autophagy is a highly conserved lysosomal degradation pathway that plays a critical role in maintaining cellular homeostasis by eliminating damaged organelles, misfolded proteins, and reactive oxygen species, thereby limiting cellular stress and genomic damage. However, in the context of cancer, autophagy exhibits a dual role—acting as a tumor suppressor during early stages of transformation while later supporting tumor cell survival under metabolic and therapeutic stress conditions [52,55,56,57]. Autophagy is also an important process in the transition from endometriosis to specific subtypes of ovarian cancers. One of the theories regarding regulation of intracellular iron assumes that iron pathway occurs via a ferritinophagic process involving NCOA4 (Nuclear Receptor Coactivator 4). NCOA4 protein remained reduced upon proteasome inhibition with autophagy inhibition, suggesting that NCOA4 is regulated in a proteasome- and autophagy-independent manner in transformed endometriotic cells [51,58,59]. Stemness-associated markers, including SOX2 and ALDH1A1, have been implicated in early HGSC development and may be associated with stem-like properties of malignant cells [53]. In this context, the term “cancer stem cells” refers to a subpopulation of malignant cells with self-renewal and differentiation capacity, rather than to normal or cancer-prone stem cells of the fallopian tube epithelium. Such stem-like tumor cells have been proposed to contribute to tumor initiation, intratumoral heterogeneity, therapeutic resistance, and disease recurrence [40,60]. However, their precise role in the initiation and progression of HGSC remains incompletely understood.

### 5.6. Alternative Low-Grade Serous Pathways

A subset of HGSCs arises from serous borderline tumors and low-grade serous carcinoma, driven by *NRAS* and secondary *TP53* mutations [40,42,52,53,54,55,56,57]. This progression is thought to reflect a stepwise model of tumor evolution, in which early activating mutations in the MAPK pathway (e.g., *NRAS*) promote cellular proliferation and the formation of borderline or low-grade lesions, while subsequent acquisition of *TP53* mutations leads to loss of genomic integrity, increased chromosomal instability, and transition to a high-grade phenotype. This model contrasts with the de novo development of most HGSCs and highlights molecular heterogeneity in their pathogenesis [9,10,43,51,59]. The comparison of the major epithelial ovarian cancer subtypes is presented in Table 2.

## 6. Alternative Precursor Lesions in Epithelial Ovarian Cancer

### 6.1. Inclusion Cysts (CIC)

Inclusion cysts (also known as CICs) are non-cancerous, thin-walled spaces in the ovarian cortex filled with fluid. The main mechanisms of CIC formation are ovulation and mechanical invagination of the ovarian surface epithelium (OSE). The surface epithelium of the ovary undergoes cyclical damage and repair under the influence of repeated ovulation. This repair process promotes invagination of the OSE into the cortex, resulting in the formation of an inclusion cyst lined by ovarian surface epithelium cells (OSE-type) [51].

Recent reports have proposed alternative theories suggesting that many ovarian tumors originate from the fallopian tube epithelium, with ciliated cells from the fallopian tube implanting into the ovarian cortex during ovulation. This mechanism posits that FTE cells can migrate and implant within the ovarian cortex, thereby forming ‘tubal-type’ CICs [51,61]. These CICs express markers characteristic of the fallopian tube epithelium, such as PAX8, rather than markers typical of OSE. CICs composed of Müllerian epithelium are potential precursors of serous tumors (both low-grade and high-grade). For high-grade tumors, the prevailing view is that they often originate from the fallopian tube epithelium. However, CICs may provide an environment in which cells from this epithelium, after acquiring mutations, transform into a tumor [11,62].

Experimental models also demonstrate that the extracellular and intracellular matrix causes fallopian tube epithelial cells (FTE) to exhibit invasive capacity, whereas OSE cells are not invasive. Pothuri et al. demonstrated that the ovarian cyst epithelium often exhibits features of aneuploidy, including increased cell proliferation and reduced apoptosis [11,42].

Furthermore, aneuploidy was present in normal-appearing cystic epithelial cells, but not superficial epithelial cells. The level of oxidative stress in the epithelial inclusion cyst was high, as evidenced by *p53* protein aggregation. In HGSC development, the process is rapid and typically occurs after acquisition of a *TP53* mutation. LGSC develops slowly, beginning with the acquisition of mutations in the *KRAS* or *BRAF* genes. CICs have been proposed as one possible substrate for the development of serous cystadenomas, which in some cases may subsequently progress to borderline serous tumors and eventually LGSC. However, the precise sequence and frequency of these transitions remain incompletely understood [11,15,42,43].

### 6.2. Ovarian Endometriosis as a Potential Precursor

Ovarian endometriosis is a well-established precursor lesion for endometriosis-associated ovarian cancers, particularly endometrioid ovarian carcinoma (ENOC) and clear cell ovarian carcinoma (CCOC), supported by molecular and histopathological evidence [25,63,64,65]. In contrast, current evidence supports a predominantly tubal origin of HGSC through the STIC pathway, and endometriosis is not recognized as a direct precursor of HGSC. Nevertheless, the chronic inflammatory and oxidative microenvironment associated with endometriosis, including iron-mediated reactive oxygen species generation, DNA damage, and activation of inflammatory pathways, could theoretically create conditions permissive to carcinogenesis [63,64]. Similar inflammatory and oxidative mechanisms may also affect the fallopian tube through retrograde menstruation, although this does not establish a biological continuum between endometriosis and HGSC [16,46,64,65,66]. Thus, endometriosis may represent a potential indirect or microenvironmental contributor to HGSC development rather than a defined precursor lesion. This hypothesis remains speculative and requires further experimental and molecular investigation.

### 6.3. Borderline Ovarian Tumors

Borderline ovarian tumors (BOTs) represent a heterogeneous group of epithelial ovarian neoplasms, accounting for 15–20% of all epithelial ovarian tumors. They are characterized by increased mitotic activity without infiltrative, destructive growth or stromal invasion, distinguishing them from invasive carcinomas. They were initially described as “semimalignant” or “low malignant potential” tumors, due to their favorable prognosis and high overall survival rate, even in advanced stages [67,68,69].

Molecular alterations observed in BOTs indicate their link to the development of type I ovarian tumors (low-grade carcinomas) according to the model proposed by Shih and Kurman. In this model ovarian tumors develop slowly and are typically preceded by benign lesions such as inclusion cysts (cystadenomas)—serous Mullerian subtype and mucinous intestinal type. Endometriotic cyst and adenofibromas are the precursors of lesions of the Mullerian subtype (mucinous endocervical/endometroid type) mucinous BOTs [10,11]. There are considered precursor lesions for low-grade ovarian carcinomas, including serous, mucinous, endometrioid, clear cell, sero-mucinous, and transitional (Brenner) tumors.

The progression from benign ovarian lesions to BOTs and subsequently to invasive low-grade carcinomas is considered a multistep molecular process driven by the gradual accumulation of genetic and epigenetic alterations. Activating mutations in the MAPK signaling pathway, particularly involving *KRAS*, *BRAF*, and *ERBB2*, are regarded as early molecular events promoting increased proliferation and survival of the epithelial cells. These alterations are frequently identified already in benign cystadenomas and persist in BOTs as well as in low-grade serous carcinomas, supporting the concept of a stepwise progression model. In contrast to high-grade ovarian carcinomas, *TP53* mutations are uncommon in BOTs, further emphasizing their distinct molecular pathogenesis [9,10,11,69].

A key distinguishing feature of BOTs from invasive carcinomas is the absence of stromal invasion. In invasive tumors, two invasion patterns are recognized: expansive or destructive. Serous BOTs have the potential to form peritoneal implants, which must not be confused with stromal invasion. Microinvasion is defined as stromal infiltration up to 5 mm in depth, without a desmoplastic stromal reaction. Peritoneal implants are frequently observed in serous BOTs. Their presence has implications for overall therapeutic management and prognosis [70,71,72,73,74].

The transition from BOTs to invasive carcinoma is thought to depend not only on additional genetic alterations but also on changes within the tumor microenvironment, including stromal remodeling, loss of cell adhesion, and acquisition of invasive potential. Increased expression of proteins involved in epithelial–mesenchymal transition (EMT), extracellular matrix degradation, and cellular motility has been associated with progression toward invasive growth. In serous BOTs, the micropapillary pattern and the presence of invasive peritoneal implants are considered morphological manifestations of enhanced biological aggressiveness and may represent intermediate stages in the evolution toward low-grade serous carcinoma [75,76,77,78,79,80,81]. Importantly, *BRCA1/BRCA2*, *RAD51C*, and *PALB2* mutations do not directly increase the risk of BOTs [79,82]. Furthermore, specific mutations in BOTs have been observed to be involved in tumorigenesis and are presented in Table 3. Recent studies also suggest that chronic inflammation, oxidative stress, and dysregulation of signaling pathways such as PI3K/AKT/mTOR and Wnt/β-catenin may contribute to the acquisition of invasive features. These findings suggest that BOTs may represent biologically dynamic lesions with variable malignant potential rather than uniformly indolent neoplasms [78,79,80,81,82].

## 7. Clinical Implications and Therapeutic Possibilities

### 7.1. Ovarian Cancer Prevention

Reduced ovulation rates, associated with hormonal contraceptive use and a higher number of pregnancies, correlate with a reduced risk of ovarian cancer not only by decreasing the fallopian tube epithelium’s exposure to reactive oxygen species (ROS) generated during ovulation but also by increasing cumulative exposure to progesterone. This observation forms the basis of the “incessant ovulation” hypothesis, which proposes that repeated cycles of epithelial injury and repair contribute to DNA damage accumulation and malignant transformation.

Several epidemiological studies have demonstrated that hormonal contraception, pregnancy, and breastfeeding are associated with a lower lifetime risk of ovarian cancer, likely due to suppression of ovulation and reduction in cumulative epithelial stress. In addition, progesterone may exert protective effects by promoting apoptosis of damaged epithelial cells and modulating local inflammatory responses [83,84,85].

These observations support the concept that reducing cumulative ovulatory injury may decrease ovarian cancer risk and highlight the importance of preventive strategies targeting early carcinogenic events within the fallopian tube epithelium.

### 7.2. Opportunistic Bilateral Salpingectomy

Opportunistic bilateral salpingectomy (OBS) with ovarian conservation has emerged as a promising strategy for reducing the risk of epithelial ovarian cancer, particularly high-grade serous carcinoma (HGSC). Its rationale is strongly supported by the tubal origin paradigm of HGSC and the identification of serous tubal intraepithelial carcinoma (STIC) as an important precursor lesion. Accordingly, removal of both fallopian tubes during an otherwise indicated benign gynecological procedure may provide an opportunity for risk reduction without removing the ovaries.

An important advantage of OBS compared with risk-reducing salpingo-oophorectomy (RRSO) is preservation of ovarian function and avoidance of premature surgical menopause. This approach may therefore be particularly relevant for women undergoing hysterectomy or other pelvic surgery who do not have an indication for ovarian removal. Current evidence suggests that salpingectomy is associated with a reduction in subsequent ovarian cancer risk; however, the magnitude of protection against HGSC specifically remains uncertain, and most available evidence is observational. OBS should therefore be considered a promising risk-reducing strategy rather than a procedure that completely eliminates ovarian cancer risk. Further prospective studies and long-term follow-up are needed to determine its effectiveness in preventing HGSC and to assess its long-term effects on ovarian function [86,87,88,89,90].

### 7.3. Current Limitations of Screening

However, ovarian cancer still remains the leading cause of death from gynecologic malignancies. The cause of the poor prognosis is the diagnosis in advanced stages, when mortality is high. Therefore, we urgently need screening tests to detect ovarian cancer at an early stage. Transvaginal ultrasound and CA-125 blood tests are widely available and commonly used to evaluate women with symptoms of ovarian cancer. However, they offer no long-term benefits in population screening and may provide adverse outcomes. Therefore, they are not recommended for women without ovarian cancer predisposition. Currently, there’s no evidence that any biomarker could detect STIC or early HGSC with acceptable sensitivity and specificity. Consequently, effective population-based screening for HGSC remains an unmet clinical need.

### 7.4. Risk-Reducing Surgery in High-Risk Populations

For women with a genetic predisposition, RRSO is a main preventive intervention, significantly reducing risk and becoming the standard of care. RRSO is recommended between the ages of 35–40 for women with pathogenic or probably pathogenic variants of *BRCA1*, and between the ages of 40–45 for carriers of *BRCA2* variants, due to rarer and later ovarian cancer diagnosis in this group. In 2023, a systematic review of 158 articles was published, which concluded that bilateral salpingo-oophorectomy in the general population can reduce the risk of ovarian cancer by approximately 80%, which, according to authors from leading American cancer centers, may translate into a 15% reduction in mortality from this cancer in the American population. The authors emphasize that the procedure does not seem to affect the risk of premature menopause. An Italian study of women diagnosed with breast cancer and a concurrent *BRCA* mutation demonstrated that prophylactic bilateral salpingo-oophorectomy significantly reduced the overall risk of death (HR, 0.40; 95% CI, 0.25–0.64; *p* <  0.001) [83,84,85,90]. The risk of death was also reduced for triple-negative breast cancer, but not for luminal B (*ERBB2*-negative). While opinions have varied over the years regarding the advisability of prophylactic salpingo-oophorectomy in women with breast cancer, this study provides strong evidence for the use of prophylactic surgery in *BRCA*-positive women already diagnosed with breast cancer [85,86,90].

The latest recommendations regarding prophylactic surgery, namely RRSO, were published in 2024 by the National Comprehensive Cancer Network (NCCN) as presented in Table 4 [91].

### 7.5. Clinical Management of STIC

The effectiveness of prophylactic surgery is assessed differently across epidemiological studies. Available data evaluating 10- and 15-year postoperative outcomes estimate the effectiveness of these procedures in preventing ovarian and endometrial cancer to be close to 100%. Current evidence indicates that in approximately 2–10% of patients undergoing prophylactic surgery, occult lesions can already be detected. These include endometrial intraepithelial neoplasia (EIN) and serous tubal intraepithelial carcinoma (STIC), as well as early-stage cancers.

Current evidence shows that diagnosis of STIC detected during SEE-FIM (a specialized histopathological examination method developed to improve the detection of early precursor lesions in the fallopian tube) in women with *BRCA* mutations was associated with a higher risk of peritoneal carcinoma, than in women without tubal lesions (10-year risk: 27.5% vs. 0.9%). Moreover, women with a *BRCA1* mutation have a lifetime risk of developing breast cancer of 45% to 80% and a lifetime risk of developing ovarian cancer of 30% to 60%. For women with a *BRCA2* mutation, the risk is 35% to 60% and 10% to 25%, respectively. Therefore, increased monitoring frequency for women with diagnosed STIC is recommended; however, no standardized follow-up protocol exists [87]. Genetic counseling and testing are also indicated for patients with no known risk who are incidentally diagnosed with STIC. Extended surgical staging according to ESGO and ESMO is recommended only in case STIC with microinvasive carcinoma, while additional routine surgical treatment remains undefined. More research is needed to develop guidelines for managing STIC [87,88,89,90].

### 7.6. Comparative Perspective Across Epithelial Ovarian Cancer Histotypes

The precursor pathways of epithelial ovarian cancer are best understood as histotype-specific rather than as manifestations of a single universal carcinogenic sequence. HGSC is most strongly linked to the fallopian tube, with p53 signatures and STIC representing the best-characterized precursor spectrum. In contrast, ENOC and CCOC show a well-established association with ovarian endometriosis, in which progressive molecular alterations, including ARID1A and PIK3CA abnormalities, may precede malignant transformation. LGSC follows a distinct, relatively indolent pathway characterized by serous borderline tumors and recurrent MAPK-pathway alterations, particularly involving KRAS and BRAF. Mucinous ovarian carcinoma appears to follow yet another pathway, often associated with benign or borderline mucinous lesions and stepwise accumulation of molecular abnormalities.

Despite these differences, several common biological processes recur across histotypes, including chronic tissue injury, oxidative stress, genomic instability, altered DNA repair, clonal selection, and remodeling of the local microenvironment. However, the initiating cell population, anatomical site, dominant driver alterations, and rate of progression differ substantially between histotypes. These differences are clinically relevant because the presence of a precursor lesion does not imply equivalent malignant potential across tumor types. Taken together, current evidence supports a model of multiple histotype-specific precursor pathways rather than a single ovarian carcinogenesis pathway. The strongest evidence for a defined precursor lesion exists for STIC in HGSC, endometriosis in ENOC and CCOC, and serous borderline tumors in the LGSC pathway. Other proposed lesions, including cortical inclusion cysts, SCE, SCOUTs, and STILs, remain biologically informative but require further validation before their independent precursor significance can be established [88,89].

## 8. Limitations

Limitations of the review process

The review process has several methodological limitations. First, the literature search was conducted in three electronic databases—PubMed/MEDLINE, Scopus, and Web of Science—and therefore relevant studies indexed in other databases or not retrieved by the predefined search strategy may have been missed. Second, the search was restricted to English-language publications, which may have introduced language-related selection bias and limited the comprehensiveness of the evidence base. The review protocol was not prospectively registered, which may limit the transparency and reproducibility of the review process. Third, the review followed a structured narrative rather than a fully systematic review methodology, and the substantial heterogeneity of the included literature prevented quantitative synthesis and statistical pooling of results. In addition, a standardized data-extraction form was not specified, and the final reviewers responsible for individual data extraction were not explicitly identified. Although screening and full-text assessment were performed independently by two reviewers, a formal study-level risk-of-bias assessment and formal assessment of publication or reporting bias were not conducted. These methodological limitations may have influenced the completeness and interpretability of the evidence synthesis and should therefore be considered when interpreting the conclusions of this review.

Limitations of the evidence

The review process itself also had several limitations. Although predefined eligibility criteria and a structured search strategy were applied, the review was conducted as a structured narrative review rather than as a fully systematic review. In addition, the authors did not specify a standardized data-extraction form, and they did not explicitly identify the reviewers responsible for the final extraction of data from individual publications. These aspects may have introduced subjectivity during study selection, data extraction, and narrative synthesis. Nevertheless, two reviewers performed screening and full-text assessment independently, resolving discrepancies through discussion and consensus.

## 9. Future Perspectives

Although STIC is currently the best-characterized precursor lesion associated with HGSC, increasing evidence indicates substantial biological heterogeneity and variable progression potential. A major priority for future research is therefore to distinguish indolent precursor lesions from those with a genuine risk of progression to invasive disease. TP53 mutation or abnormal p53 expression alone is unlikely to provide sufficient prognostic information. Instead, future studies should focus on integrated biomarkers incorporating chromosomal instability, aneuploidy, HR deficiency, telomere dysfunction, proliferative activity, and microenvironmental features. Aneuploidy-based approaches, including RealSeqS and the REAL-FAST classifier, represent promising strategies for molecular risk stratification, but require prospective validation before clinical implementation.

Molecular risk assessment should increasingly combine histopathological and genomic information. Spatial and multimodal profiling may be particularly valuable because precursor lesions are small and biologically heterogeneous. However, most current evidence is derived from retrospective or cross-sectional studies, limiting the ability to determine whether specific molecular alterations truly predict progression. Prospective longitudinal studies with standardized pathological assessment and long-term follow-up are therefore essential to establish clinically meaningful predictors of malignant transformation.

Artificial intelligence-assisted pathology may further improve the detection and characterization of subtle precursor lesions. Machine-learning approaches could support recognition of morphological abnormalities, quantitative assessment of p53 and Ki-67 patterns, and integration of histological features with molecular information. However, current AI applications in ovarian pathology remain limited by relatively small datasets, inter-institutional variability, and insufficient external validation. Future studies should therefore prioritize large multicenter datasets and prospective validation before AI-based tools are incorporated into routine diagnostic practice.

Liquid biopsy represents another potentially important direction for early detection. Circulating tumor DNA, cell-free DNA, extracellular vesicles, circulating RNA, and protein signatures could provide minimally invasive biomarkers of early disease. Nevertheless, detecting microscopic precursor lesions such as STIC may be substantially more difficult than detecting established HGSC because of their low tumor burden. At present, no circulating biomarker has demonstrated sufficient sensitivity and specificity for reliable detection of STIC or early HGSC. Future studies should therefore evaluate highly sensitive multimodal approaches and determine whether circulating molecular signals can complement tissue-based risk assessment. The ultimate goal of precursor research is early detection and cancer prevention. However, detecting every molecularly abnormal lesion may result in overtreatment if many precursor lesions remain biologically indolent. Future prevention strategies should therefore focus on identifying lesions at highest risk of progression and tailoring surveillance or intervention accordingly. This may be particularly important in genetically predisposed populations, in whom risk-reducing surgery remains an established preventive strategy, while the optimal management and follow-up of incidentally detected STIC remain incompletely defined [92,93,94,95].

Overall, future research should move from descriptive characterization of precursor lesions toward precision risk stratification and cancer interception. Integrating molecular biomarkers, aneuploidy profiling, computational pathology, liquid biopsy, and clinical risk factors may ultimately allow identification of precursor lesions with the highest malignant potential. However, prospective multicenter validation will be essential before these approaches can be translated into routine clinical practice.

## 10. Conclusions

This review summarizes the established and emerging precursor pathways involved in ovarian carcinogenesis, distinguishing evidence-based concepts from hypotheses that require further investigation. Ovarian cancer develops through histotype-specific pathways driven by distinct molecular alterations. High-grade serous carcinoma arises predominantly through the tubal carcinogenesis pathway involving *p53* signatures and serous tubal intraepithelial carcinoma (STIC), although increasing evidence supports biological heterogeneity and variable progression potential among these precursor lesions. Other histological subtypes originate from alternative precursor lesions, including endometriosis-associated lesions and cortical inclusion cysts. Advances in molecular profiling, particularly aneuploidy-based classifiers such as REAL-FAST, may improve risk stratification of precursor lesions and facilitate earlier detection and prevention. However, these approaches remain investigational and require prospective validation in large multicenter studies before they can be incorporated into routine clinical practice.

Importantly, the presence of a precursor lesion does not necessarily indicate inevitable malignant progression, particularly in the case of biologically heterogeneous STIC lesions. Future research should therefore focus on identifying molecular and morphological features that distinguish high-risk precursor lesions from indolent lesions, integrating genomic profiling, artificial intelligence-assisted pathology, and minimally invasive biomarkers. Such approaches may ultimately enable histotype-specific risk stratification and support earlier detection and more individualized prevention of epithelial ovarian cancer.

## Figures and Tables

**Table 1:** Histopathological and molecular features of major fallopian tube epithelial alterations implicated in serous carcinogenesis.

Lesion	Morphology	Immunophenotype	Molecular Alterations	Typical Location	Current Evidence as a Precursor Lesion
p53 signature	Morphologically bland secretory cells, typically with enlarged or hyperchromatic nuclei but without significant cytologic atypia or architectural disturbance.	Aberrant p53 expression; typically low proliferative activity.	TP53 mutation is the defining molecular alteration; usually few additional genomic abnormalities.	Predominantly distal/fimbrial fallopian tube.	Well-established early molecular alteration associated with the HGSC pathway, but not sufficient for malignant transformation.
Secretory cell expansion (SCE)	Localized expansion/proliferation of morphologically relatively bland secretory epithelial cells.	Secretory-cell phenotype; immunophenotypic profile may overlap with normal tubal secretory epithelium.	Limited molecular evidence; relationship to TP53-driven precursor lesions remains incompletely defined.	Variable distribution along the fallopian tube.	Biological significance remains uncertain; should not currently be considered an established HGSC precursor.
SCOUT	Localized proliferation/expansion of secretory cells, generally without the marked cytologic atypia characteristic of STIC.	Typically associated with secretory epithelial markers; PAX2 expression may help distinguish SCOUTs from some other lesions.	Molecular alterations are heterogeneous and generally distinct from the canonical TP53-driven STIC pathway.	More frequently identified in proximal regions of the fallopian tube, although distribution may be broader.	Not established as an obligatory or early precursor of HGSC; likely represents a biologically distinct secretory-cell alteration.
STIL	Atypical secretory epithelial proliferation with cytologic abnormalities, but without unequivocal features of STIC.	Often shows abnormal p53 expression and increased proliferative activity compared with p53 signatures.	TP53 alterations may be present; genomic abnormalities are variable.	Predominantly distal/fimbrial region.	Intermediate/controversial lesion; may represent a spectrum of atypical tubal epithelial changes, but its independent precursor significance remains uncertain.
STIC	Marked cytologic atypia, nuclear enlargement/hyperchromasia, loss of polarity, epithelial stratification, and increased mitotic activity.	Aberrant p53 expression and increased Ki-67 proliferative activity; tubal secretory phenotype.	TP53 mutation is nearly universal; associated with increasing chromosomal instability and additional genomic alterations.	Strong predilection for distal/fimbrial tube and tubal-peritoneal junction.	Strongest clinicopathological, molecular, and experimental evidence among tubal precursor lesions for the development of HGSC; progression is not necessarily inevitable.

**Table 2:** Comparison of the major epithelial ovarian cancer subtypes. The table summarizes differences between high-grade serous carcinoma (HGSC), low-grade serous carcinoma (LGSC), endometrioid ovarian carcinoma (ENOC), and clear cell ovarian carcinoma (CCOC) regarding their precursor lesions, cells of origin, key molecular alterations, genomic instability, signaling pathways, tumor evolution, and clinical behavior. HGSC is characterized by nearly universal *TP53* mutations and high genomic instability, whereas LGSC is primarily driven by MAPK pathway alterations. ENOC and CCOC are closely associated with endometriosis and frequently harbor alterations in the PI3K/AKT/mTOR pathway, particularly *ARID1A* and *PIK3CA* mutations. These molecular differences contribute to distinct patterns of tumor progression, treatment response, and prognosis.

Feature	HGSC	LGSC	ENOC	CCOC
Main precursor	STIC (fallopian tube epithelium)	Serous borderline tumor (BOT)	Endometriosis/adenofibroma	Endometriosis
Cell of origin	Fallopian tube epithelium (secretory cell lineage)	Serous tubal epithelium	Müllerian endometrium-like epithelium	Endometriotic epithelium
Early key driver	*TP53* mutation (>95%)	*KRAS*, *BRAF*, *NRAS*	*PTEN* loss, *ARID1A* mutation	*ARID1A* loss, *PIK3CA* mutation
DNA repair defects	*BRCA1/2*, HR deficiency	Rare	Moderate	Moderate
Genomic instability	Very high	Low	Intermediate	Intermediate-high
Cell cycle disruption	*CCNE1* amplification	Rare	Occasional *CDKN2A*, *CTNNB1*	PI3K axis-driven
*TP53* status	Nearly universal mutation	Usually wild-type early	Variable	~50%
MAPK pathway	Not primary	Central driver	Secondary	Secondary
PI3K/AKT/mTOR	Subsets	Rare	Central	Very important
Inflammation	STIC stress	Low	High (endometriosis)	Very high (ROS/iron)
Telomeres	Crisis + telomerase escape	Gradual shortening	Moderate	Short + oxidative stress
Evolution	Rapid STIC→HGSC	Stepwise BOT→LGSC	Stepwise endometriosis	Stepwise endometriosis
Clinical behavior	Aggressive	Indolent	Intermediate	Aggressive + chemoresistant
Hallmark	*TP53* + CIN (chromosomal instability)	MAPK	PI3K + hormones	*ARID1A* + ROS

**Table 3:** Selected molecular alterations in borderline ovarian tumors (BOTs) [80,81,82,83,84,85,86].

Marker	Biological Function	Expression Pattern in BOTs/Comparison	Clinical Significance
*ERBB2* (HER2)	Member of the EGFR family; activates PI3K/AKT and MAPK/ERK pathways regulating proliferation, migration, and differentiation	Dysregulated activation in BOTs	Contributes to tumorigenesis and cell proliferation
Claudin-1	Tight junction protein involved in cell adhesion, signal transduction, and paracellular transport	Overexpression observed in BOTs	Associated with peritoneal implants and micropapillary pattern; unfavourable prognostic factors
Rb2/p130 (Retinoblastoma protein family)	Cell cycle regulation (G1/S checkpoint control)	Benign: nuclear; BOTs: mixed nuclear–cytoplasmic; malignant: predominantly cytoplasmic	In mucinous BOTs, low nuclear and high cytoplasmic expression correlates with increased risk of progression; potential diagnostic and prognostic marker
Aurora A kinase	Centrosomal kinase regulating G2/M transition, mitotic spindle formation, and cell division	BOTs show intermediate expression pattern between benign (nuclear) and malignant (cytoplasmic)	Overexpression linked to poor prognosis; involved in NF-κB signaling and cell cycle regulation; potential therapeutic target

**Table 4:** NCCN version 2.2024 outlining recommended management following the detection of genetic mutations.

Gene	Ovarian Cancer Risk (%)	Recommendation
*BRCA1*	38–58%	Risk-reducing salpingo-oophorectomy at age 35–40 depending on reproductive plans; hysterectomy may also be considered due to serous endometrial cancer risk
*BRCA2*	13–29%	Risk-reducing salpingo-oophorectomy at age 40–45 depending on reproductive plans
*RAD51C*	10–15%	Recommend risk-reducing salpingo-oophorectomy at age 45–50
*RAD51D*	10–20%	Recommend risk-reducing salpingo-oophorectomy at age 45–50
*BRIP1*	5–15%	Recommend risk-reducing salpingo-oophorectomy at age 45–50
*PALB2*	3–5%	Consider risk-reducing salpingo-oophorectomy at age 45–50
*ATM*	2–3%	Individualized approach depending on other risk factors
*MLH1*	4–20%	Individualized approach depending on other risk factors
*MSH2/EPCAM*	8–38%	Individualized approach depending on other risk factors
*MSH6*	<1–13%	Individualized approach depending on other risk factors; insufficient data
*PMS2*	1.3–3%	Individualized approach depending on other risk factors; no clear increased risk

## Data Availability

The data extracted from the included studies are available from the corresponding author upon reasonable request.

## Supplementary Materials

The following supporting information can be downloaded at: https://www.mdpi.com/article/10.3390/cancers18183036/s1.
